# The near‐global mesospheric potassium layer: Observations and modeling

**DOI:** 10.1002/2015JD023212

**Published:** 2015-08-07

**Authors:** E. C. M. Dawkins, J. M. C. Plane, M. P. Chipperfield, W. Feng

**Affiliations:** ^1^ School of ChemistryUniversity of LeedsLeedsUK; ^2^ School of Earth and EnvironmentUniversity of LeedsLeedsUK

**Keywords:** potassium layer, mesospheric metal, satellite retrieval

## Abstract

The meteoric metal layers act as unique tracers of chemistry and dynamics in the upper atmosphere. Existing lidar studies from a few locations show that K exhibits a semiannual seasonality (winter and summer maxima), quite unlike the annual seasonality (winter maximum and summer minimum) seen with Na and Fe. This work uses spaceborne observations made with the Optical Spectrograph and InfraRed Imager System instrument on the Odin satellite to retrieve the near‐global K layer for the first time. The satellite data (2004 to mid‐2013) are used to validate the implementation of a recently proposed potassium chemistry scheme in a whole atmosphere chemistry climate model, which provides a chemical basis for this semiannual seasonal behavior. The satellite and model data show that this semiannual seasonality is near global in extent, with the strongest variation at middle and high latitudes. The column abundance, centroid layer height, and root‐mean‐square width of the K layer are consistent with the limited available lidar record. The K data set is then used to investigate the impact of polar mesospheric clouds on the metal layers at high latitudes during summer. Finally, the occurrence frequency of sporadic K layers and their possible link to sporadic *E* layers are examined.

## Key Points

- First study of the global K layer using satellite data to validate a new model
- Evidence of removal of K by polar mesospheric clouds
- Determination of the global occurrence frequency of sporadic K layers

## 1 Introduction

There has been much interest in the upper atmospheric metal layers since 1929, when the American astronomer Vesto Slipher first observed bright yellow radiation at 589 nm in the night sky spectrum. A decade later, this was confirmed as being the result of Na resonance by *Cabannes et al*. [1938] and *Bernard* [1939], with a source located in the terrestrial atmosphere. Since those early days, much progress has been made in the observation and understanding of these mesospheric metal layers, through a combination of ground‐based lidar observations, laboratory studies, and atmospheric modeling [e.g., *Feng et al*., 2013; *Marsh et al*., 2013], and, more recently, satellite observations [e.g., *Fan et al*., 2007a, 2007b; *Fussen et al*., 2004, 2010; *Dawkins et al*., 2014; *Langowski et al*., 2014, 2015].

To date the majority of observations still come from ground‐based metal resonance lidar observatories, with a particular focus on Na and Fe, primarily due to their use as tracers for both upper atmospheric wind and temperature [e.g., see *Fricke and von Zahn*, 1985; *Bills and Gardner*, 1993; *Gardner et al*., 2001; *She et al*., 2004]. In contrast, very few measurements have been made of the K layer, with permanent lidar stations based at only two locations: Arecibo (18°N, 293°E) and Kühlungsborn (54°N, 12°E) [e.g., see *Friedman et al*., 2002, 2003, 2013; *Raizada et al*., 2004; *Gerding et al*., 1999; *Höffner and Friedman*, 2004; *Höffner and Lübken*, 2007].

Of these observable meteoric metals, currently available data indicate that the K layer exhibits a markedly different seasonal pattern from the other metals [e.g., see *Eska et al*., 1998; *Gerding et al*., 2000; *Dawkins et al*., 2014; *Plane et al*., 2014]. While the Fe, Na, Ca, and Mg layers exhibit dominant annual variations (with early winter maxima and midsummer minima at midlatitudes), the K layers exhibit semiannual variations with a summertime maximum which exceeds that of the wintertime [*Gerding et al*., 2000; *Plane*, 2003]. This contrasting behavior is particularly surprising as both Na and K are Group 1 (Alkali) metals and thus might be expected to exhibit similar behaviors.

An explanation for this semiannual seasonal behavior has recently been proposed by *Plane et al*. [2014] who showed that it arises via two key differences in the neutral and ion chemistry of the two species. The neutral K chemistry has no analogous reaction to the NaHCO_3_ + H → Na + H_2_CO_3_ which recycles the Na reservoir back to Na; the activation energy for the K reaction is too large to be efficient within the relatively cold temperatures of the mesosphere/lower thermosphere (MLT) region. This means that the only way that the main K reservoir species, KHCO_3_, can be converted back to neutral K is via photolysis, which renders the metal chemistry of the underside of the layer temperature independent, unlike that of Na. Additionally, the K^+^ ion is larger than Na^+^, so it can form weakly bound clusters only during the very low summer MLT temperatures. In contrast, Na^+^ clusters exist throughout the year. These weakly bound K^+^ clusters can then undergo dissociative recombination with electrons to yield neutral K, resulting in a summertime K maximum not seen in Na.

This work employs the Odin/Optical Spectrograph and InfraRed Imager System (OSIRIS) K retrieval algorithm first described in *Dawkins et al*. [2014] to provide a more detailed description of the near‐global K layer, both in terms of the mean monthly column density, layer width and height, and also to explore some applications of this new K data set. Specifically, our aims are to assess how well the National Center for Atmospheric Research (NCAR) Whole Atmosphere Community Climate Model with potassium chemistry (WACCM‐K) [*Plane et al*., 2014; *Feng et al*., 2015] can model the near‐global K layer and to use both satellite and model data to examine the latitude‐specific differences in the K layer characteristics and how these differ from the Na layer. We then examine the impact of polar mesospheric clouds (PMCs) on the K layer and the geographic occurrence of sporadic K layers and their correlation to sporadic *E* layer occurrence.

## 2 Data

The near‐global K data use an optimal estimation technique to retrieve K number density profiles from dayglow measurements made by the Optical Spectrograph and Infrared Imager System (OSIRIS) spectrometer on board the Sun‐synchronous and polar‐orbiting Odin satellite. These density profiles have typical layer peak errors of ±15% and a 2 km vertical grid resolution [*Dawkins et al*., 2014]. The OSIRIS K data set is available from 2004 to present and has a latitude coverage extending from ±82°. As the K measurements rely on sunlight, only limited coverage is available in the winter hemisphere at middle to high latitudes.

NCAR's WACCM is a comprehensive coupled chemistry climate model and is part of the Community Earth System Model framework. The standard model with specified dynamics extends from the Earth's surface up to ~140 km with 88 vertical levels (vertical resolution of approximately 1.5 km in the lower atmosphere and ~3.5 km in the MLT region) and a horizontal resolution of 1.9° × 2.5° (latitude‐longitude). WACCM contains a fully interactive chemistry scheme and includes shortwave heating and photolysis from extreme UV to Lyman *α*. Above 60 km, it incorporates non‐local‐thermal‐equilibrium IR transfer. It includes a parameterization for gravity waves from convection and fronts (and their subsequent breaking within the mesopause region). Metal chemistry modules have been added for Na [*Marsh et al*., 2013], Fe [*Feng et al*., 2013], Mg [*Langowski et al*., 2015], and K [*Plane et al*., 2014]. For this study, the meteorological reanalyses used to specify the dynamics in WACCM are the European Centre for Medium‐Range Weather Forecasts (ECMWF) Re‐Analysis (ERA)‐Interim [see *Dee et al*., 2011].

## 3 Results and Discussion

### 3.1 The Near‐Global K Layer Observed by OSIRIS

**Figure 1:**
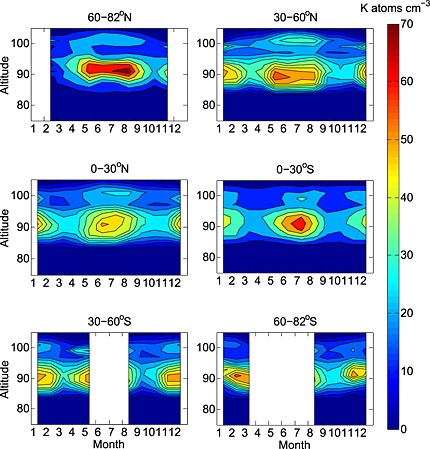
The zonally averaged variation of the K layer measured by the OSIRIS spectrometer as a function of month and altitude and binned into different latitude bands. Multiyear mean (2004–2013). Altitudes (ordinate axis) are in kilometers, and the color bar is K in atom cm^−3^.

Figure 1 illustrates monthly vertical seasonal profiles of the OSIRIS K layer, zonally averaged in a series of latitude bins. Each monthly average comprises all data within ±2 weeks of the fifteenth day of the month. The largest seasonal variation occurs at latitudes poleward of 60° in both hemispheres. This is consistent with satellite observations of Na [*Fussen et al*., 2004, 2010; *Fan et al*., 2007a, 2007b; *Hedin and Gumbel*, 2011] and modeling studies of both Na [*Marsh et al*., 2013] and Fe [*Feng et al*., 2013]. This may be a result of transport of K atoms poleward from low latitudes as a result of the meridional circulation, as first identified by *Gardner et al*. [2005] who sought to explain the unusually high‐wintertime lidar‐observed densities of Na and Fe at the South Pole. The Northern and Southern Hemisphere 60°–82° profiles show local summertime minima below 90 km between May to August and November to March, respectively; this is likely a result of polar depletion by polar mesospheric clouds (PMCs), as discussed in section 3.4. The treatment of multiple scattering and the careful subtraction of the background from the pure K emission signal mean that the presence of PMCs are not thought to complicate the OSIRIS retrieval of K.

In all profiles, there is a pronounced secondary layer at around 100 km. These layers are likely to be sporadic K layers (see section 3.5) with sufficiently high concentrations and occurrence frequencies that they still appear in the monthly averaged data.

**Figure 2:**
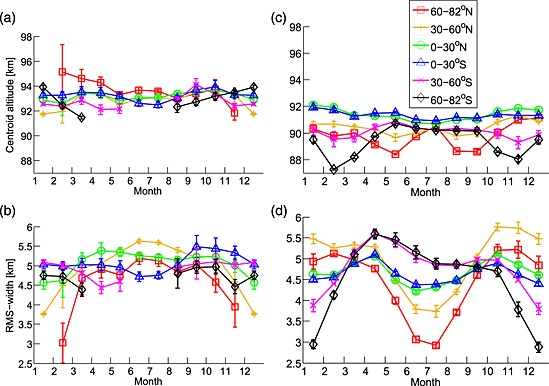
Characteristics of the retrieved and modeled K layers as a function of month for different latitude bands zonal mean centroid altitude for (a) OSIRIS and (b) WACCM‐K; zonal mean RMS width for (c) OSIRIS and (d) WACCM‐K. The vertical bars represent the mean error.

A comparison of the mean centroid altitudes of the zonal mean OSIRIS K layer profiles is presented in Figure 2a. The mean global geometric centroid height, $\bar{z}$ is determined as

$$\bar{z}=\frac{\sum z_{i}F\left(z_{i}\right)}{\sum F\left(z_{i}\right)}$$

where *F*(*z_i_*) is the K number density at a given altitude, *z_i_*.

The mean global centroid height is approximately 93.1 km, with a relatively small mean variation of ±0.6 km throughout the year (<1% of the mean centroid height). The centroid altitude variation is typically greatest at high latitudes (93.6 ± 0.9 km for 60–82°N, 92.9 ± 0.9 km for 60–82°S) and monotonically decreases toward low latitudes (93.2 ± 0.4 km for both the 0–30°N and 0–30°S profiles). Due to the limited vertical resolution of the OSIRIS K retrieval (~2 km), the centroid altitude cannot be located to the same resolution and precision as a ground‐based lidar. Indeed, the variability of the mean OSIRIS centroid altitude is typically smaller than the vertical resolution of the retrieval. Despite this caveat, the semiannual variation (highest centroid altitudes during the equinoxes) is largely consistent with lidar measurements of the K layer at Arecibo and Kühlungsborn [*Eska et al*., 1998; *Friedman et al*., 2002; *Höffner and Lübken*, 2007]. The corresponding mean global centroid altitudes of the zonal mean WACCM‐K profiles are presented in Figure 2b. The mean global centroid altitude of 90.4 km is lower than the corresponding retrieved value (93.1 km), but the mean global variation is the same at ±0.6 km. WACCM‐K does not currently include PMCs; the effect of such implementation will be the subject of future work.

The root‐mean‐square (RMS) layer width profiles for different latitude bands are shown in Figure 2c. The RMS layer width is determined as follows:

$$RMS=\sqrt{\left(\frac{\sum F\left(z_{i}\right)z_{i}^{2}}{\sum F\left(z_{i}\right)}-{\bar{z}}^{2}\right)\text{.}}$$

The mean global OSIRIS RMS width is 4.9 km with an absolute mean variation of ±0.4 km (derived from an approximate absolute range of 3.0–5.6 km). This lies comfortably within the 2.3–5.8 km range (mean = 4.0 km) recorded during the Polarstern shipborne campaign [*Eska et al*., 1999], which consisted of 66 nights of lidar measurements made between March and July 1996 across a latitude transect of 71°S to 54°N.

The vertical resolution of the OSIRIS K retrieval results in only a relatively limited sensitivity to changes in the RMS width, in addition to the centroid altitude. In general, however, the global OSIRIS RMS widths compare well to the available lidar observations and show latitudinal seasonal variation of the RMS width in both hemispheres. The largest annual variation is seen at high northern latitudes (±0.7 km for both 60–82°N and 30–60°N, respectively, compared to approximately ±0.2 km for the other latitude bands). Care must be taken during the interpretation of the satellite results due to both the limited vertical retrieval resolution and the scarcity of data during the high‐latitude polar night periods. The corresponding WACCM‐K zonal mean RMS widths are presented in Figure 2d; the global mean of 4.7 ± 0.6 km (absolute range: 4.4–5.0 km) compares well to both the OSIRIS mean RMS width as well as to the Polarstern campaign data. Relatively little seasonal variation at low latitudes is supported by the findings of *Friedman et al*. [2002] and *Fricke‐Begemann et al*. [2002] who reported seasonal variations at Arecibo (18°N) and Tenerife (28°N), respectively.

For latitudes between 0 and 30°, the late spring/early summer OSIRIS RMS width is typically ~0.3 km larger than during winter, which is in general agreement with the findings of *Friedman et al*. [2002]. The corresponding low‐latitude WACCM‐K profiles exhibit both clear spring and fall maxima. At higher latitudes, the OSIRIS RMS‐width profiles exhibit a more complex seasonal behavior. Both the 30–60°N and 60–82°N OSIRIS profiles exhibit similar summertime RMS‐width maximum and wintertime minimum behaviors (note that the 60–82°N profile does not contain data for January and December). The corresponding middle and high southern latitude profiles do not exhibit as much seasonal variation as these northern profiles. In contrast to the OSIRIS observations, the 60–82°N WACCM‐K profiles exhibit well‐defined summertime RMS‐width minima and wintertime maxima, which is in closer agreement to the observations of *Eska et al*. [1998] who found that the RMS width was larger in winter than during summer for Kühlungsborn at 54°N. The sensitivity of the RMS width to the topside and bottomside of the mean profiles (where the OSIRIS‐retrieved profiles typically have the greatest associated errors) can make the identification of causal mechanisms difficult. The wintertime dynamical downwelling, which occurs at middle‐high latitudes, acts to compress the K layer which can result in reduced wintertime RMS widths, which may explain the OSIRIS 30–60°N and 60–82°N profiles. The RMS width is also particularly sensitive to the occurrence of sporadic K layers which exhibit a semiannual variation (summer and wintertime maxima, see section 3.5). Both of these effects may result in the summertime RMS‐width maxima seen within these same latitude band profiles. It should be noted that the OSIRIS February data for 60–82°N exhibit both a greatly increased centroid altitude, yet greatly reduced RMS width, relative to the rest of the seasonal layer. This likely arises as a result of the general spring/fall K column density minima, coinciding with relatively few OSIRIS measurements during this period, and the undue influence of sporadic K layers in certain profiles on the monthly mean. The complex interaction of PMC activity, the general meridional circulation and sporadic K layers, and their influence on the observed and modeled layer width characteristics will be the focus of future work.

### 3.2 The Modeled K Layer

In a previous paper [*Plane et al*., 2014], a new K chemistry scheme was added to WACCM, and the output compared to lidar data from Kühlungsborn at 54°N. We now use the OSIRIS K data to test the performance of this new chemistry scheme on a near‐global scale.

**Figure 3:**
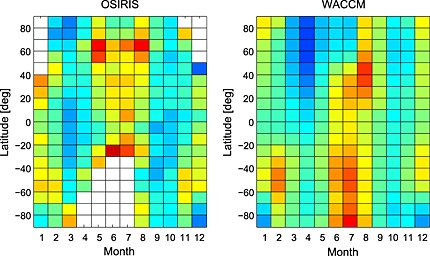
Comparison of the monthly averaged K column density (units: atom cm^−2^) as a function of month and latitude (2004–2013): (left) OSIRIS‐retrieved K densities; (right) WACCM‐K model data.

The OSIRIS and WACCM K column abundance as a function of month and latitude is compared in Figure 3. In both cases, data from 2004 to 2013 are averaged to remove any solar cycle effects. The model is able to simulate the semiannual behavior of the K column abundance reasonably well. Hemispheric asymmetry is seen in both the satellite and model data sets with a stronger seasonal variation typically observed in the Northern Hemisphere; similar results have also been seen in the long‐term OSIRIS Na data set reported in *Hedin and Gumbel* [2011] and is likely attributed to the hemispheric differences in the relative sizes of the Arctic and Antarctic polar vortices and the impact that these have on global circulation. The overall reasonable agreement of the WACCM modeled K layer with the OSIRIS data, at nearly all latitudes, supports the new K chemistry scheme outlined in *Plane et al*. [2014]. The first climatology of the global K layer is presented in the supporting information.

### 3.3 The Differential Response of Modeled K and Na to Temperature

**Figure 4:**
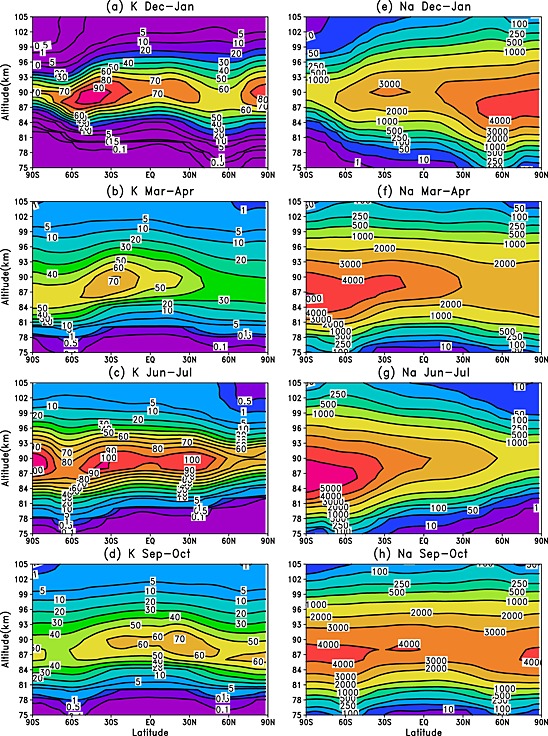
Comparison of the K and Na zonal mean monthly mean concentration profiles (units: atom cm^−3^), using WACCM‐K and WACCM‐Na with ERA‐Interim data (2004–2013), as a function of altitude and latitude.

**Figure 5:**
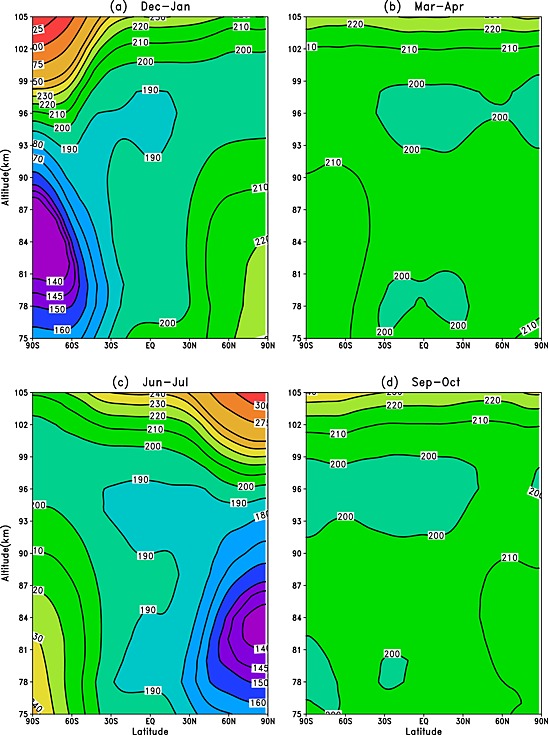
WACCM with ERA‐Interim modeled zonal‐mean monthly‐mean absolute temperature profiles (2004–2013), as a function of altitude and latitude.

The differences between the WACCM modeled Na and K seasonal variations are examined further in Figure 4 with the corresponding WACCM temperature profiles shown in Figure 5. During the solstice months (December–January and June–July), the Na density displays a maximum in the winter hemisphere only, with the largest densities at middle and high latitudes which correspond to the occurrence of the warmest MLT temperatures. Within the summer hemisphere, where the MLT is at its coldest, the Na layer is depleted (more Na remains in the reservoir NaHCO_3_ due to the temperature dependence of the NaHCO_3_ + H → Na + H_2_CO_3_ reaction). In contrast, the K layer exhibits maxima in both the summer and winter hemispheres, even during the very cold summer polar region. In the spring/fall months (March–April and September–October), the behavior of the Na and K layers is reasonably similar; both metals exhibit a Southern Hemisphere maximum during March–April and a less pronounced hemispheric difference during September–October.

**Figure 6:**
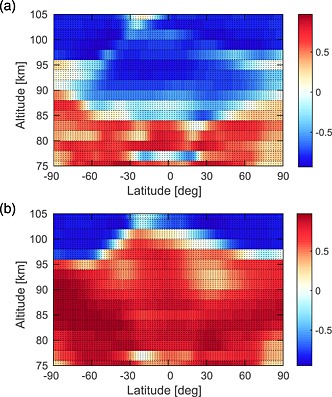
Correlation analyses between (a) WACCM‐K number density and temperatures and (b) WACCM‐Na number density and temperature, for all months between 2004 and 2009, as a function of latitude and altitude. The independent variable is time. The color scale represents the correlation coefficient, *r*.

A correlation analysis between both WACCM monthly mean metal data sets and temperature has been performed across 2004–2013 (for the December–January, March–April, June–July, and September–October periods), as a function of latitude and altitude, and is shown in Figure 6. Outside the tropics, Na shows a clear positive correlation with temperature at all latitudes below 95 km, largely because of the positive activation for the NaHCO_3_ + H reaction. In contrast, there is a strong anticorrelation above this height, because the ion‐molecule chemistry which converts Na^+^ to Na is faster at lower temperature. These correlations have been well documented in other studies, such as *Plane et al*. [1999] and *Fan et al*. [2007a, 2007b]. The K layer exhibits the same negative correlation above 95 km. However, below this height K demonstrates a rather more complicated relationship with temperature, with weak positive or negative correlations at different latitudes. This arises because the neutral K chemistry does not have a strong temperature dependence, so that dynamical effects (e.g., downwelling/upwelling at high latitudes) become more pronounced.

### 3.4 Polar Depletion of the K Layer

The very low temperatures (<150 K) within the summer polar mesosphere support the growth of polar mesospheric clouds (PMCs) [*Lübken*, 1999] over a wide latitude band (typically poleward of 55°) at altitudes between 82 and 88 km [e.g., *Lübken and Höffner*, 2004; *DeLand et al*., 2006] and with a mean PMC altitude of ~83 km in the Northern Hemisphere (~2 km higher in the Southern Hemisphere) [*von Cossart et al*., 1997; *Wickwar et al*., 2002; *Alpers et al*., 2001; *Fiedler et al*., 2003; *Höffner et al*., 2003; *Thayer et al*., 2003; *Thayer and Pan*, 2006]. PMCs typically appear from mid‐May to late August in the NH, reaching a peak brightness approximately 20 days after the summer solstice [*Olivero and Thomas*, 1986; *DeLand et al*., 2006].

The PMC ice surfaces efficiently remove metal atoms at very low temperatures [*Murray and Plane*, 2005] and this removal leads to summertime minima as documented in the Na and Fe metal layers [e.g., see *Gardner et al*., 1988; *Plane et al*., 2004; *Gardner et al*., 2005; *She et al*., 2006]. Although the K^+^ ion chemistry results in a summertime maximum which appears to be unique to this metal, lidar observations at Spitsbergen (78°N) have demonstrated that the K layer exhibits a local minimum during PMC season [*Lübken and Höffner*, 2004; *Raizada et al*., 2007].

**Figure 7:**
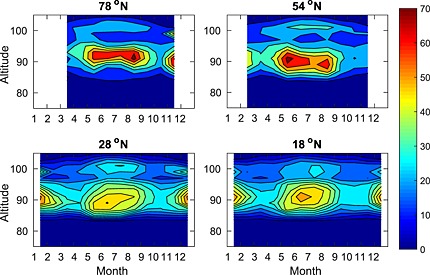
The seasonal mean OSIRIS K layer at latitudes corresponding with the Spitsbergen (78°N), Kühlungsborn (54°N), Tenerife (28°N), and Arecibo (18°N) lidar stations, all ±5°.

**Figure 8:**
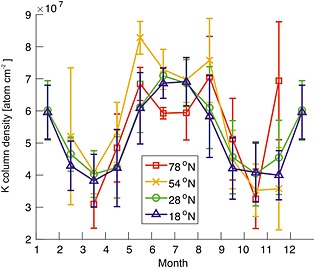
Comparison of zonal mean OSIRIS K layer column density centered around Spitsbergen (78°N), Kühlungsborn (54°N), Tenerife (28°N), and Arecibo (18°N), all ±5°. Vertical bars indicate the error of the mean (2σ) (units: K atom cm^−2^).

Satellite observations of the K layer provide a means for detecting the occurrence of PMCs and their impact on the K layers globally. The seasonal variation of the K layer for four different latitude bands is shown in Figure 7. At 78°N, the summertime K layer exhibits depletion on the underside between May and August, which is not apparent in the low‐latitude layers at 28°N and 18°N. The maximum K depletion occurs within June/July when polar mesospheric temperatures are lowest and the atmospheric upwelling is strongest, transporting water vapor up to these altitudes. Along with the increased rate of ionization during the summer (which results in a greater fraction of neutral K being converted to K^+^ via charge transfer with NO^+^ and O_2_^+^), depletion by PMCs below 85 km can also affect the topside of the K layer because of increased downward transport of K atoms from higher altitudes through vertical eddy diffusion [*Raizada et al*., 2007]. This effect can be seen in the 78°N profile, with a reduction of K atoms on both the topside and bottomside of the layer. The depletion of the K layer, most likely caused by PMCs, is investigated in further detail in Figure 8. Both the 78°N profile and, to a lesser extent, the 54°N profile exhibit local minima in the column density during May–August, which is consistent with removal of metal atoms by PMCs on the underside.

### 3.5 Occurrence of K Sporadic Layers

Sporadic metal layers were first reported by *Clemesha et al*. [1978] who detected thin Na layers which appeared overhead the lidar station at São José dos Campos (23°S, 46°W). These layers exhibited peak densities 2.5 to 3 times larger than the typical Na layer, and after appearing suddenly, the events typically had a duration of between only a few minutes and several hours. A variety of mechanisms have been proposed to explain sporadic layers, including a possible link to meteor showers [*Clemesha et al*., 1978], the sputtering of metal atoms from cosmic dust particles [*von Zahn et al*., 1987; *Beatty et al*., 1989], the redistribution of the background metal layers by gravity waves [*Kirkwood and Collis*, 1989; *Delgado et al*., 2012], the dissociation of metal reservoir species such as NaHCO_3_ [*von Zahn and Murad*, 1990], temperature fluctuations which produce an enhancement from some unknown metal reservoir [*Zhou et al*., 1993], and finally the neutralization of metal ions in a descending sporadic *E* layer [*von Zahn and Hansen*, 1988; *Hansen and von Zahn*, 1990; *Kane and Gardner*, 1993; *Cox and Plane*, 1998]. This last theory, which followed the observation of a spatial and temporal correlation between sporadic *E* layers and sporadic neutral metal layers, and has also been supported by a combination of laboratory [e.g., *Cox et al*., 2001] and modeling studies [*Heinselman*, 2000; *Collins et al*., 2002] is now widely accepted as being the major mechanism (but not perhaps the only mechanism) involved in the formation of sporadic layers.

**Figure 9:**
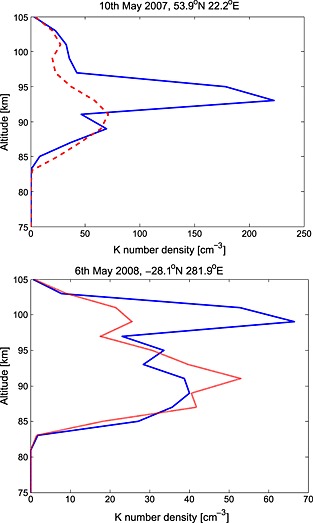
Examples of sporadic K layers detected by OSIRIS (blue solid line). The standard mean K layer (red broken line) is derived from the appropriate monthly zonal average for latitudes within ±5° of the possible sporadic K layer profile.

To assess the sporadic K (hereafter termed K_s_) occurrence probability using the OSIRIS near‐global data, it is important to first choose appropriate criteria for defining a sporadic layer. Here we follow the criteria used by *Fan et al*. [2007a, 2007b], which were first proposed by *Clemesha* [1995]. A K_s_ layer is positively identified if it fulfills the following criteria: the density of the possible sporadic layer exceeds 3 times the density of the standard mean K density layer at the same altitude (for the same latitude band) and the full width half maximum (FWHM) of the possible sporadic layer is smaller than 4 km. The second criterion was chosen to be compatible with the vertical resolution of the retrieved data (2 km) and ensures that only possible sporadic layers with the required narrow FWHM are positively identified. Example sporadic layers from OSIRIS profiles which fit these criteria are presented in Figure 9, which shows that K_s_ layers can occur at different altitudes and with very different concentrations.

**Figure 10:**
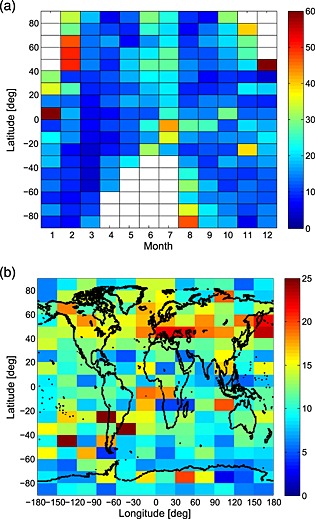
(a) Percentage occurrence from 2004 to 2011 of OSIRIS‐detected sporadic K layers as a function of month and latitude (10° bins); (b) percentage occurrence of OSIRIS‐detected sporadic K layers (2004–2011) as a function of latitude (10° bins) and longitude (30° bins).

Figure 10a presents a global map of the probability of K_s_ occurrence as a function of month and latitude (in 10° bins). The data in each grid box represent the percentage occurrence of possible sporadic layers between 2004 and 2011. It is important to emphasize that the occurrence frequency is for the local times of the OSIRIS measurements, i.e., about 0600 and 1800 LT. Nevertheless, in agreement with the nighttime observations reported in *Friedman et al*. [2002] the highest K_s_ occurrence frequencies occur in summer and winter, with spring/summer minima. There appear to be very high K_s_ frequency occurrences (>50%) at Northern Hemisphere midlatitudes (40–70°N) during February. Additionally, very high occurrences (>60%) are present in January between 0 and 10°N, and December between 40 and 50°N.

Figure 10b presents the mean percentage occurrence of K_s_ as a function of latitude (in 10° bins) and longitude (in 30° bins) for the same observation period. The data show geographically very variable percentage K_s_ occurrences, with an apparent slight Northern Hemisphere bias in the number of K_s_ events, particularly between 40 and 70°N. Additionally, there are also regions of high‐percentage occurrence around the lower half of South America and the Atlantic and Pacific regions either side; this enhancement effect was also seen in the Na_s_ results of *Fan et al*. [2007a, 2007b]. Those authors also noted that there was a particularly high Na_s_ occurrence in the Southern China and Japan regions. Although the OSIRIS K_s_ data do not show relatively high enhancements in this region, there is a region of locally very high (>22%) occurrence northeast of Japan (40–60°N, 150–180°E). The slight Northern Hemisphere bias seen in the OSIRIS K_s_ data contrasts with the Southern Hemisphere bias reported in the Na_s_ data by *Fan et al*. [2007a, 2007b]. In addition, the global mean occurrence of the Ks is approximately 12% which contrasts to the 5% occurrence probability of Na_s_. The reasons for these differences will be the subject of future work.

As discussed earlier, the occurrence of sporadic *E* layers (*E*_s_) represents the most probable cause of sporadic metal layers. A tentative comparison can be made between the OSIRIS K_s_ data and that of the Constellation Observing System for Meteorology, Ionosphere and Climate GPS radio occultation measurements of *E*_s_ data presented in *Chu et al*. [2014]. During December–May there is a prominent occurrence of *E*_s_ over South America, extending across both the southern Atlantic and Pacific regions. This corresponds well to the region of relatively enhanced K_s_ activity shown in Figure 10b. In March–August there is a region of relatively high *E*_s_ occurrence centered over eastern Asia (encompassing Japan, extending up to approximately 60°N), mainland Europe and North Africa (10–60°N), and over North America/Canada (>15°N). Each of these corresponds to “hot spot” regions of relatively high K_s_ occurrence within the OSIRIS data. This provides support to the leading theory that sporadic metal layers occur as a result of neutralization of metal ions in descending sporadic *E* layers. Within the solstice profiles (June–August and December–February), the *E*_s_ occurrence rate maximizes within the summer hemisphere. While this would explain the summertime maximum in the Northern Hemisphere OSIRIS K_s_ occurrence data relative to the equinoxes, the equivalent Southern Hemisphere summer maximum is not seen. Additionally, at the majority of latitudes the OSIRIS data show a stronger winter maximum in K_s_ occurrence than in summer. These differences may be explained by changes in tidal descent rates and related changes in the atomic O density, since O controls the rate of conversion of the metal ions into neutral atoms [*Cox et al*., 2001].

## 4 Summary and Conclusions

The unusual behavior of K, compared with the other metals, has been a long‐standing problem, first postulated during the 1970s. A new theoretical basis developed by *Plane et al*. [2014] for this behavior produces a modeled response for seasonal and diurnal variations [*Plane et al*., 2014; *Feng et al*., 2015], which compares well to the K observations. The work presented here confirms this theory using satellite data and has established for the first time that this semiannual seasonality is near global in extent, with the strongest variation at middle and high latitudes. The column abundance, centroid layer height, and RMS width of the K layer appear to be consistent with the limited lidar studies available. The removal of K atoms on the underside of the layer during summer at high latitudes (>50°), almost certainly caused by uptake on PMC ice particles, is seen in the satellite data. The occurrence of K_s_ compares well with available lidar statistics at Arecibo (18°N), and the near‐global satellite record shows a semiannual variation. The positive correlation between the geographic occurrence of the sporadic K and sporadic *E* layers supports the leading theory that such sporadic metal layers occur as a result of neutralization of metal ions in descending sporadic *E* layers.

## Supporting information



Figure S1

Table S1

Text S1
