# Supplementary material for: The near‐global mesospheric potassium layer: Observations and modeling
Source: J Geophys Res Atmos. 2015 Aug 7;120(15):7975–87. doi: 10.1002/2015JD023212 (PMC4949710; doi:10.1002/2015JD023212)
Supplement: Supplementary file 1 — Figure S1 [file JGRD-120-7975-s001.pdf]

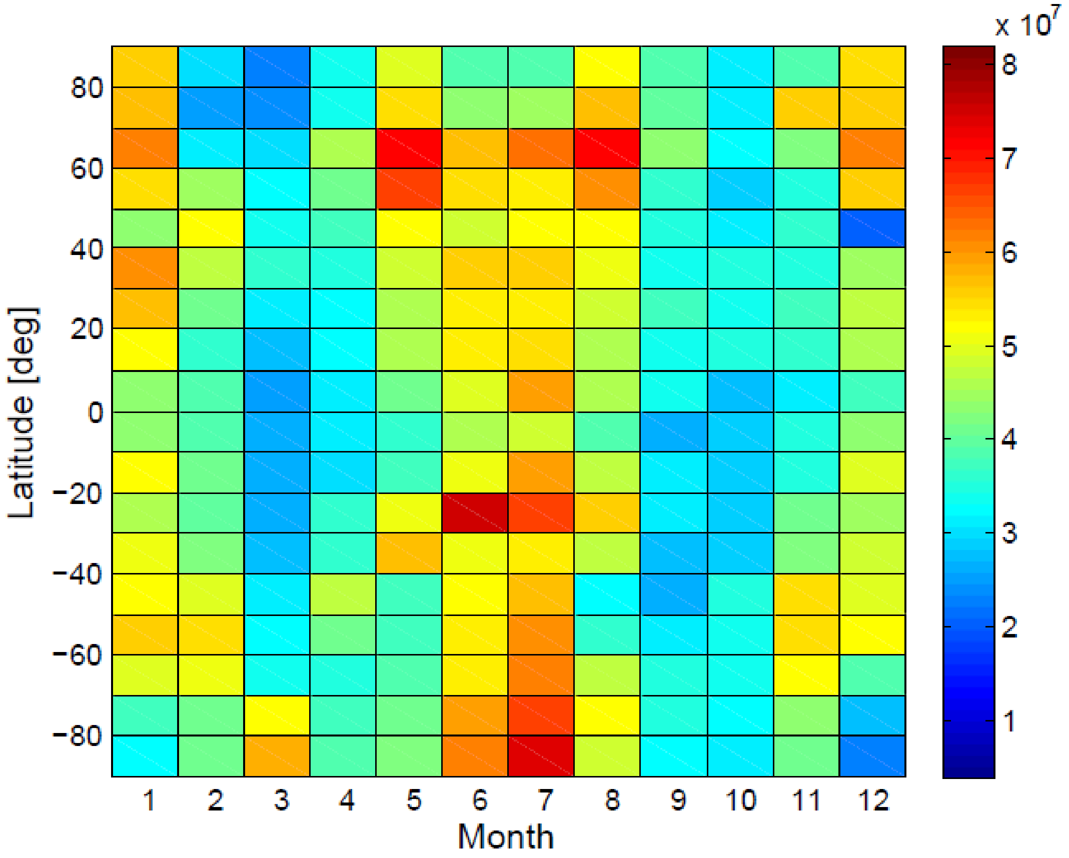

Figure S1. Global climatology of the K column density, consisting of OSIRIS data supplemented by WACCM data within the winter polar regions.
