# Supplementary material for: The near‐global mesospheric potassium layer: Observations and modeling
Source: J Geophys Res Atmos. 2015 Aug 7;120(15):7975–87. doi: 10.1002/2015JD023212 (PMC4949710; doi:10.1002/2015JD023212)
Supplement: Supplementary file 2 — Table S1 [file JGRD-120-7975-s002.pdf]

	Jan	Feb	Mar	Apr	May	Jun	Jul	Aug	Sep	Oct	Nov	Dec
80-90°N	5.61	3.02	2.28	3.34	4.98	3.89	3.83	5.18	3.91	3.17	3.86	5.49
70-80 ^{O}N	5.73	2.52	2.35	3.39	5.42	4.40	4.45	5.70	3.94	3.14	5.58	5.61
60-70 ^{O}N	6.19	3.20	2.96	4.62	7.13	5.71	6.26	7.12	4.35	3.23	4.19	6.17
50-60 ^{O}N	5.49	4.50	3.21	4.09	6.64	5.49	5.31	6.12	3.66	2.96	3.51	5.61
40-50 ^{O}N	4.31	5.27	3.44	3.81	5.21	4.87	5.18	5.23	3.53	3.15	3.62	1.99
30-40 ^{O}N	6.10	4.67	3.59	3.49	4.90	5.54	5.58	5.06	3.38	3.47	3.47	4.52
20-30 ^{O}N	5.76	4.13	3.17	3.24	4.64	5.39	5.32	4.81	3.72	3.44	3.74	4.74
10-20 ^{O}N	5.23	3.63	2.79	3.21	4.63	5.37	5.50	4.66	3.42	3.45	3.68	4.64
0-10 ^{O}N	4.42	3.86	2.59	3.17	4.17	5.01	5.89	4.55	3.35	2.80	3.15	3.79
0-10 ^{O}S	4.37	3.82	2.64	3.12	3.65	4.57	4.88	3.93	2.62	2.87	3.52	4.34
10-20 ^{O}S	5.24	4.11	2.69	3.04	3.76	5.12	5.93	4.74	3.09	2.88	3.51	5.02
20-30 ^{O}S	4.60	3.97	2.64	3.68	5.03	7.49	6.73	5.52	3.15	2.89	4.18	4.54
30-40 ^{O}S	5.14	4.27	2.80	3.62	5.73	5.10	5.29	4.73	2.78	2.87	4.29	4.85
40-50 ^{O}S	5.23	4.96	3.20	4.72	3.78	5.26	5.67	3.22	2.71	3.47	5.51	4.97
50-60 ^{O}S	5.56	5.47	3.25	4.12	3.80	5.39	6.06	3.61	3.12	3.33	5.46	5.27
60-70 ^{O}S	4.92	5.04	3.35	3.49	3.85	5.40	6.20	4.70	3.47	3.39	5.25	3.83
70-80 ^{O}S	3.80	4.12	5.21	3.70	4.10	5.91	6.70	5.19	3.49	3.24	4.32	2.78
80-90 ^{O}S	3.26	4.07	5.85	3.84	4.29	6.22	7.36	4.81	3.24	3.14	4.17	2.24

Table S1. Global climatology of the K column density, consisting of OSIRIS data supplemented by WACCM data within the winter polar regions. All values represent the K column density in units: 10^7 atoms cm^{-2}.
