# Supplementary material for: The near‐global mesospheric potassium layer: Observations and modeling
Source: J Geophys Res Atmos. 2015 Aug 7;120(15):7975–87. doi: 10.1002/2015JD023212 (PMC4949710; doi:10.1002/2015JD023212)
Supplement: Supplementary file 3 — Text S1 [file JGRD-120-7975-s003.pdf]

Journal of Geophysical Research

Supporting Information for

# The near-global mesospheric potassium layer: Observations and modeling.

E.C.M. Dawkins[1,2], J.M.C. Plane[1*], M.P. Chipperfield[2], W. Feng[1,2]

1. School of Chemistry, University of Leeds, UK

2. School of Earth and Environment, University of Leeds, UK

* Corresponding author (j.m.c.plane@leeds.ac.uk)

## Contents of this file

Text S1

Figure S1

Table S1

## Introduction

This supporting information provides the first global K climatology and consists of OSIRIS data supplemented by scaled WACCM-ERA data within middle and high latitude winter regions.

**Text S1.**

The first global K climatology is presented in Figure S1 and the associated column density values are provided in Table S1. The data consists predominantly of monthly OSIRIS K column density data (integrated over 75-105 km) zonally averaged into 10$^{o}$

latitude bins. As the OSIRIS instrument records a useable signal during daylight conditions only, there is limited coverage in the winter hemisphere at middle and high latitudes. Within these regions, the OSIRIS data is supplemented by scaled WACCM-ERA K data. This model data is scaled; where both OSIRIS and WACCM data exist, a mean ratio between the two is calculated and used to scale the model data within the same latitude bands during which there is no OSIRIS coverage.
